# Methylation profile of a satellite DNA constituting the intercalary G+C-rich heterochromatin of the cut trough shell *Spisula subtruncata* (Bivalvia, Mactridae)

**DOI:** 10.1038/s41598-017-07231-7

**Published:** 2017-07-31

**Authors:** Daniel García-Souto, Brankica Mravinac, Eva Šatović, Miroslav Plohl, Paloma Morán, Juan J. Pasantes

**Affiliations:** 1 Departamento de Bioquímica, Xenética e Inmunoloxía, Universidade de Vigo, E-36310 Vigo, Spain; 2 Division of Molecular Biology, Ruđer Bošković Institute, Bijenička cesta 54, 10000 Zagreb, Croatia

## Abstract

Tandemly repeated DNAs usually constitute significant portions of eukaryotic genomes. In bivalves, however, repetitive DNAs are habitually not widespread. In our search for abundant repetitive DNAs in trough shells, we discovered a novel satellite DNA, SSUsat, which constitutes at least 1.3% of the genome of *Spisula subtruncata*. As foreseen by the satellite DNA library hypothesis, we confirmed that this satellite DNA is also present in two other Mactridae species, showing a highly conserved nucleotide sequence together with a dramatic diminution in the number of repeats. Predominantly located at the G + C-rich intercalary heterochromatin of *S. subtruncata*, SSUsat displays several DNA methylation peculiarities. The level of methylation of SSUsat is high (3.38%) in comparison with bivalve standards and triplicates the mean of the *S. subtruncata* genome (1.13%). Methylation affects not only the cytosines in CpG dinucleotides but also those in CHH and CHG trinucleotides, a feature common in plants but scarce and without any clear known relevance in animals. SSUsat segments enriched in methylated cytosines partly overlap those showing higher sequence conservation. The presence of a chromosome pair showing an accumulation of markedly under-methylated SSUsat monomers additionally indicates that the methylation processes that shape repetitive genome compartments are quite complex.

## Introduction

Along recent years, the application of molecular genetic techniques to the study of marine organisms has supplied a plethora of information on the structure and evolution of bivalve genomes^1–3^. In accordance with data from other eukaryotes in which repetitive sequences constitute an important fraction of the genome^4^, it has been estimated that in some bivalves about one third of the genomes is represented by repetitive sequences^2, 3^. In spite of that, our knowledge about repetitive sequences in bivalves, as well as in other organisms, is still fragmentary, mainly due to technical difficulties in their sequencing, assembling and annotation^2, 3^.

Satellite DNAs (satDNAs), constituted by arrays of tandem head-to-tail DNA repeats, are paramount among repetitive sequences and can be located in centromeric, intercalary and/or subtelomeric chromosomal regions^5, 6^. While classical experimental methods relying on restriction endonuclease digestion of genomic DNA are only able to detect the most abundant satellites in a genome, modern approaches based on Next Generation Sequencing (NGS) followed by bioinformatic analyses via specialized software, such as RepeatExplorer^7^, enable high-throughput searches for satDNAs. The use of NGS has revealed that eukaryotic genomes can harbour many different satellite families, constituting the satellitome of a species^8^. For example, NGS approaches have unveiled 37 different satDNAs in the satellitome of the wood rush *Luzula elegans* ^9^ and 62 in that of the grasshopper *Locusta migratoria* ^8^. Though many different satellites dissimilar in both length and sequence may coexist within a single genome, monomers from the same family may display low sequence divergence within a species due to concerted evolution^5^. However, after reproductive isolation and in absence of selective pressure, mutations may be differentially homogenized and fixed among lineages, resulting in the outcome of new specific satDNA variants. In this way, closely related species sharing a set (a library) of satDNAs may show a differential spreading of some of these variants across their genomes due to divergent contraction or amplification events^10, 11^. The library model can explain the observation that some satDNAs are overrepresented in one species but either absent or present in low copy numbers in closely related taxa^12–20^.

Although it has been proposed that satDNAs can act as heterochromatin modulators, participating in gene expression control and play a role in the dynamics of centromere assembly^21–23^, the real functions of satDNAs still remain unknown and the epigenetic mechanisms operating on them are far from understood. As happens in other invertebrates^24^, the genomes of bivalves studied until now show highly methylated tracts, mostly corresponding to genes, interspersed with methylation depleted areas in intergenic regions^25, 26^. Invertebrate taxa are also characterized by presenting a rather sparse methylated repetitive DNA fraction, ranging from low to almost negligible depending on the species^25, 27, 28^, contrastingly to vertebrates and plants. In regards to bivalves, the oyster *Crassostrea gigas* presents unmethylated long terminal repeats (LTRs) and long interspersed elements (LINEs), whereas short interspersed elements (SINEs) show a certain degree of methylation that reaches the mean genomic level for satDNAs^29^. Additionally, as revealed by the use of restriction enzymes sensitive to methylation, the DTF2 satellite of the wedge shell *Donax trunculus* displayed methylation^30, 31^. The differences in the methylation behaviour of different types of sequences in bivalves do not suggest a mere DNA silencing role for DNA methylation in these organisms, therefore reinforcing the possibility of alternative functions within invertebrate genomes^27, 32^.

In the past four decades, the use of base-specific fluorochromes as chromomycin A3 (CMA) and 4′,6-diamidino-2-phenylindole (DAPI) to stain metaphase chromosomes have presented a distinction between G + C-rich and G + C-poor chromosome bands in many organisms. In bivalves, G + C-rich regions are relatively scarce, mostly coincident with nucleolus organizing regions (NORs)^33–38^. Exceptions to this pattern are the wedge shell *Donax trunculus* ^30, 39^, the zebra mussel *Dreisena polymorpha* ^40, 41^ and the trough shells *Mactra stultorum* and *Spisula subtruncata* ^42^ showing many G + C-rich bands non-coincident with the NORs. Furthermore, C-banding demonstrated that these regions were heterochromatic in both wedge shells^30^ and trough shells^42^. The molecular composition of this G + C-rich heterochromatin is still unknown. In a previous work, we mapped the abundant (2%), G + C-rich DTF2 satDNA that exhibits CpG methylation to subtelomeric regions of many *D. trunculus* chromosomes^30^; in most cases being far away from the predominantly intercalary, G + C-rich heterochromatin.

In this study we have recorded a novel satDNA, conserved within the examined species of the bivalve family Mactridae, which is the main component of the G + C-rich heterochromatin in the cut trough shell *Spisula subtruncata*. The methylation status of the *S. subtruncata* satellite was explored by both bisulfite genomic sequencing and immunocytological detection and the results were compared with those of the whole genome, as detected by methylation-sensitive amplification polymorphism (MSAP) and ELISA assays.

## Results

### Characterization of SSUsat

**Figure 1:**
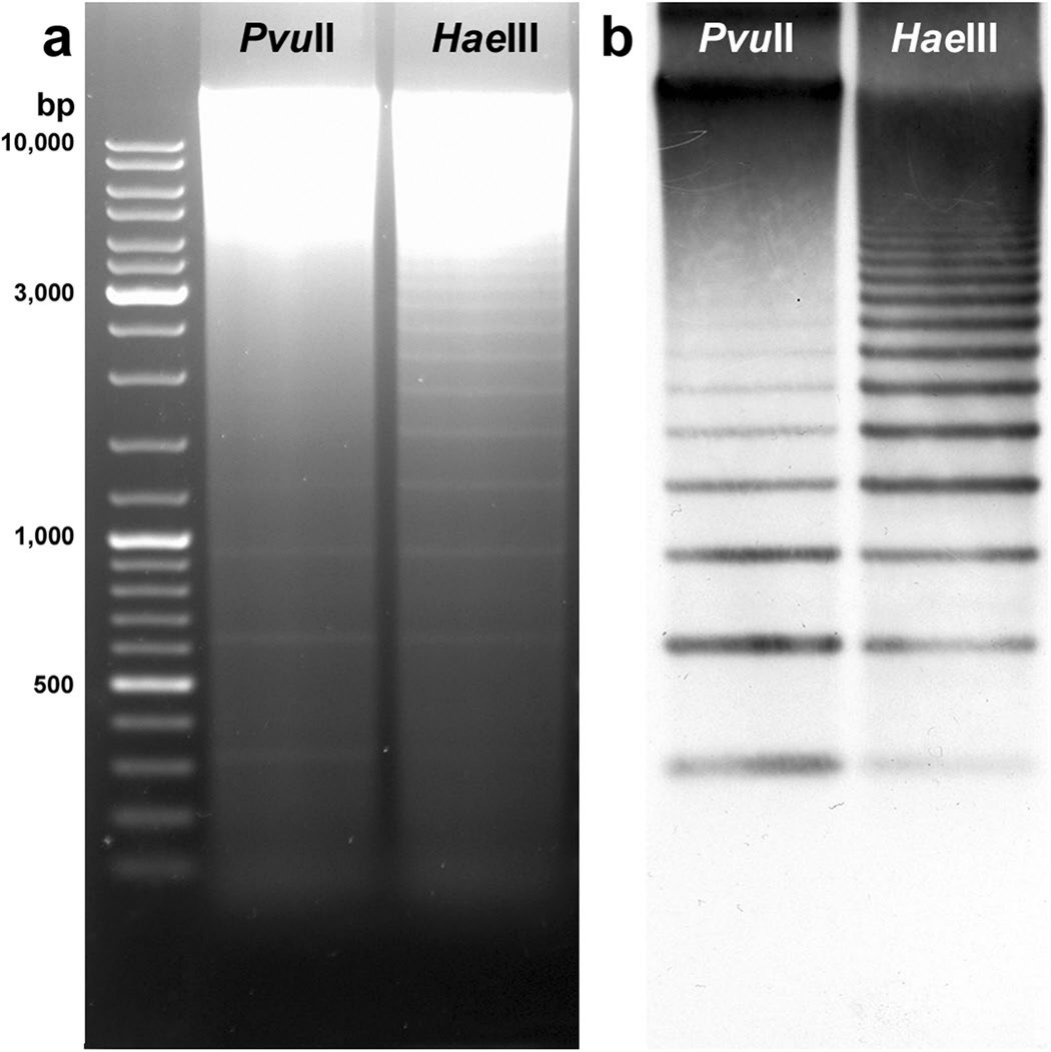
Southern blot hybridisation analysis of SSUsat repeats in *Spisula subtruncata*. Agarose gel electrophoresis of *Pvu*II and *Hae*III digested genomic DNA of *Spisula subtruncata* showing ladder-like multimer bands of a 315 bp monomer unit (**a**). After being Southern blotted on a nitrocellulose membrane, the electrophoresed DNA was hybridised with an SSUsat monomer probe yielding identical ladder-like multimer bands of a 315 bp monomer unit (**b**).

Partial digestions of *Spisula subtruncata* genomic DNA with *Pvu*II and *Hae*III REs yielded identical ladder-like multimer bands of a 315 bp monomer unit (Fig. 1a), indicative of the presence of a satellite DNA in the genome of *S. subtruncata*. Southern blotting, using the 315 bp fragment recovered from the *Hae*III digestion as a probe, gave signals on all bands of both *Hae*III and *Pvu*II ladders, with multimers distinctive up to several kb (Fig. 1b). This regularity of the ladder signals is consistent with the presence of an abundant satellite DNA presumably organized into long arrays within the *S. subtruncata* genome. The same approach did not reveal any signal in either of the other two Mactridae species (*Spisula solida* and *Mactra stultorum*) nor in a species of the related family Donacidae (*Donax trunculus*) (data not shown).

Dot blot analysis performed at high stringency conditions (68 °C) showed that these repeats accounted for at least 1.3% of the *S. subtruncata* genome (Supplementary Fig. 1a). Medium (65 °C) and low (60 °C) stringent dot blots indicated that related, less homologous sequences are also abundant (2.5 to 4%) in *S. subtruncata* (Supplementary Fig. 1b,c), suggesting that a large number of repeats related to SSUsat coexist within the cut trough shell genome. This approach however did not detect homologous sequences in the genomes of *S. solida*, *M. stultorum* and *D. trunculus* (Supplementary Fig. 1a,b,c).

**Figure 2:**
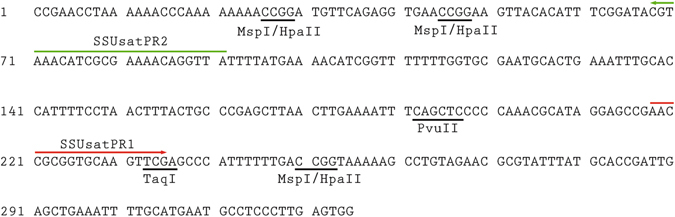
Consensus sequence of the SSUsat monomers from *Spisula subtruncata*. On the basis of the DNA sequences of the recovered SSUsat monomers from *Spisula subtruncata* genome, a consensus sequence was derived. Restriction sites for *Msp*I/*Hpa*II, *Pvu*II and *Taq*I are underlined. Green and red arrows indicate the positions of PCR primers used for SSUsat amplification in related species.

DNA fragments recovered from the gel bands corresponding to putative *S. subtruncata* monomers, dimers, trimers and tetramers were cloned and sequenced. The SSUsat representative sequence was deposited in the NCBI GenBank database under the accession number KY657249. The SSUsat consensus sequence is displayed in Fig. 2 (see Supplementary Fig. 2 for the alignment of the 39 monomeric sequences obtained from 10 monomers, 6 dimers, 3 trimers, and 2 tetramers). 38 out of 39 monomers were 315 bp long, and only one monomer showed a single nucleotide deletion. In addition to the monomer length conservation, 39 monomeric sequences also showed a low overall nucleotide diversity (Pi = 0.0294), having G + C-content of 44.05%. Similarity analysis of SSUsat monomeric sequences derived from the cloned multimers did not reveal any form of higher order repeat organization.

The SSUsat consensus sequence was blasted against NCBI GenBank database, but no significant similarities were found using megablast and discontiguos megablast algorithms.

By querying SSUsat consensus sequence through Repbase^43^, a database that contains a collection of different types of known repetitive sequences and mobile elements, significant hits (at least >15% query coverage) were not found. As neither of the searches yielded significant matches, we conclude that SSUsat represents a novel satellite DNA sequence, not previously described within any other species. In addition, local blast searches against *Crassostrea gigas* genome and partial genomic libraries of other bivalve species (*Donax trunculus*, *Ruditapes decussatus, Ruditapes philippinarum*) also demonstrate that SSUsat is a novel satDNA.

To confirm that the 39 SSUsat monomer variants analyzed here were accurate representatives of those present in the genome of *S. subtruncata*, Southern blotting was performed on genomic DNA digested with restriction enzymes characterized by presenting single cleavage sites situated in either variable or conserved regions of the SSUsat. *Hae*III and *Taq*I, single cutters with conserved restriction sites on all sequenced monomers, gave restriction profiles showing a prominent 315 bp band accompanied with few multimers (Supplementary Fig. 3). In contrast, *Pvu*II, a single cutter showing targets in a variable region, ergo only in a reduced proportion of the monomers (Supplementary Fig. 2), showed a prominent high molecular weight smear corresponding to uncut, target-free multimers (Supplementary Fig. 3). Additionally, the methylation status of the sequence was inferred by using *Hpa*II and *Msp*I, isoschizomeres whose restriction targets (three on SSUsat) only differ on cytosine methylation. The presence of the same sub-monomeric size fragments in both digestions indicates that majority of the SSUsat monomers are not methylated at the target positions. However, the slightly more intensive hybridisation signal observed in the *Msp*I digestion reaction (Supplementary Fig. 3b) suggests that a certain portion of SSUsat monomers could be subject to methylation.

### Sequence conservation of SSUsat

**Figure 3:**
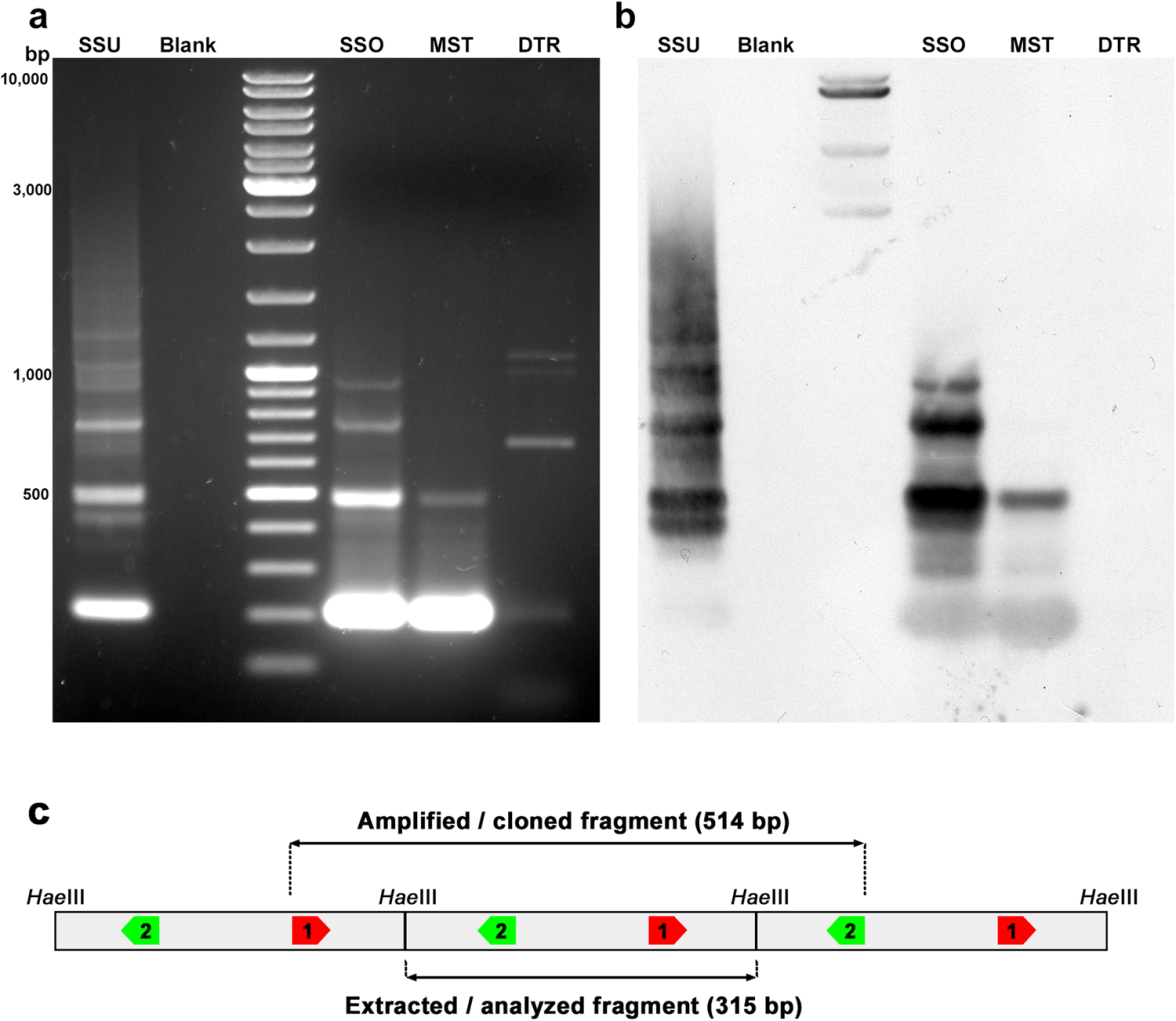
PCR amplification and Southern blot hybridisation of SSUsat in different bivalve species. Agarose gel electrophoresis of the SSUsat fragments obtained after PCR amplifying genomic DNA of *Spisula subtruncata* (SSU), *Spisula solida* (SSO), *Mactra stultorum* (MST) and *Donax trunculus* (DTR) using primers derived from the SSUsat consensus sequence **(a)**. Corresponding Southern blot hybridisation using an SSUsat probe (**b**). Blank represents PCR reaction without DNA template. (**c**) Scheme of tandemly organized SSUsat monomers showing the locations of the inversely orientated PCR primers (SSUsatPR1 in red, SSUsatPR2 in green).

Since Southern blot and dot blot assays failed to detect the presence of SSUsat in related species, we applied a PCR assay. Based on the alignment of *S. subtruncata* SSUsat monomers (Supplementary Fig. 2) and presumable tandem organization of SSUsat in related species, a pair of PCR primers in the reverse orientation was designed (Fig. 2, Fig. 3c). SSUsat was then successfully amplified by PCR in both *S. solida* and *M. stultorum* (Fig. 3a). In addition to the most prominent fragments of ~200 bp, the reversely oriented primers also amplified ~500 bp long fragments, therefore suggesting that at least some of the SSUsat sequences in *S. solida* and *M. stultorum* are organized in tandem of at least three successive monomers (Fig. 3c). Southern blotting using as a probe labelled SSU monomeric variants from *S. subtruncata*, revealed that SSUsat homologous sequences were indeed present in both species (Fig. 3b). On the other hand, no specific PCR amplification was obtained in distantly related *D. trunculus*, indicating that *D. trunculus* lacks target sequences similar enough to be amplified with the selected primers (Fig. 3). In other words, there is possibility that SSUsat sequence were either highly divergent or completely absent from the *D. trunculus* genome. 315-bp long, full-size monomers (21 from *S. solida*, and 11 from *M. stultorum*) were excised from ~500 bp PCR-fragments (Fig. 3c), and used in further analyses.

**Figure 4:**
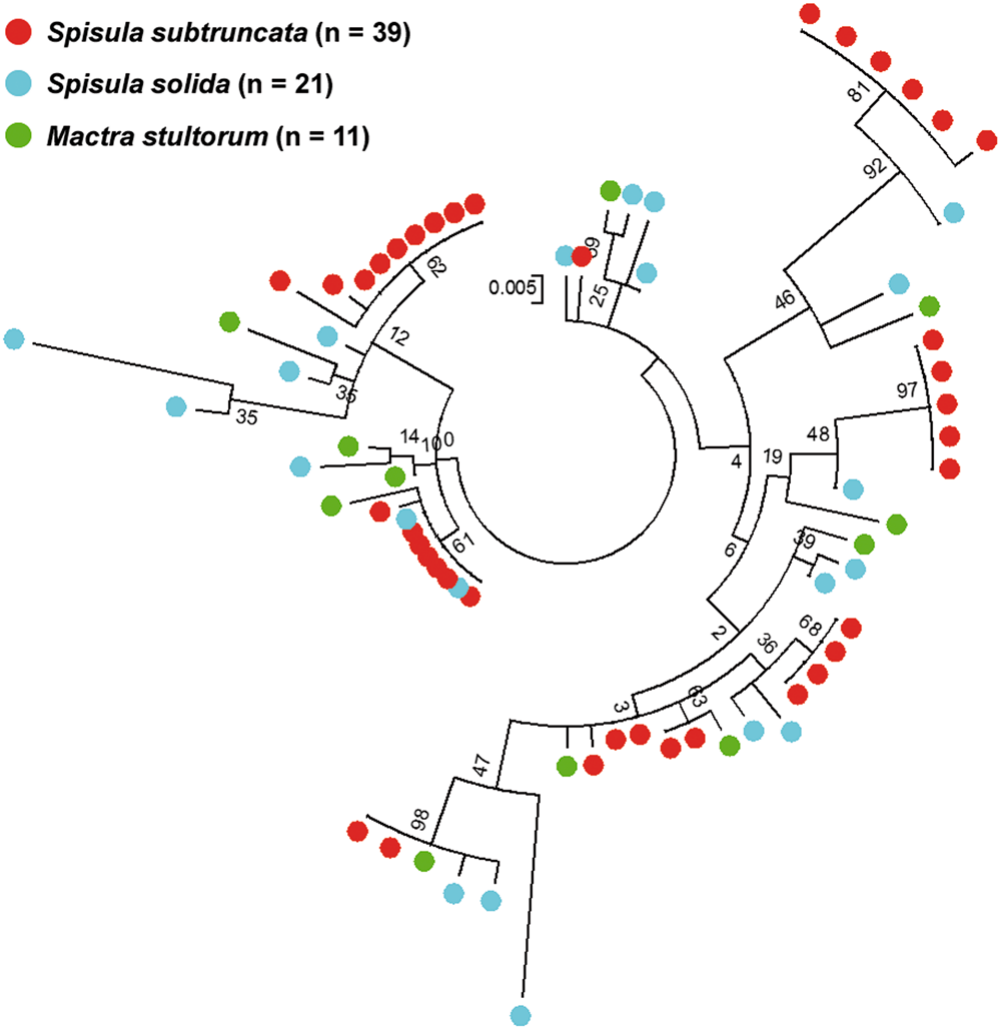
SSUsat maximum likelihood tree. Maximum likelihood tree built by Kimura 2-parameter model with Gamma distribution on the basis of the 71 SSUsat monomeric sequences isolated from *Spisula subtruncata* (39 monomers), *Spisula solida* (21) and *Mactra stultorum* (11) and 500 bootstrap replicates. The tree is based on the alignment shown in Supplementary Fig. 2. Numbers in internal nodes indicate bootstrap support values.

**Figure 5:**
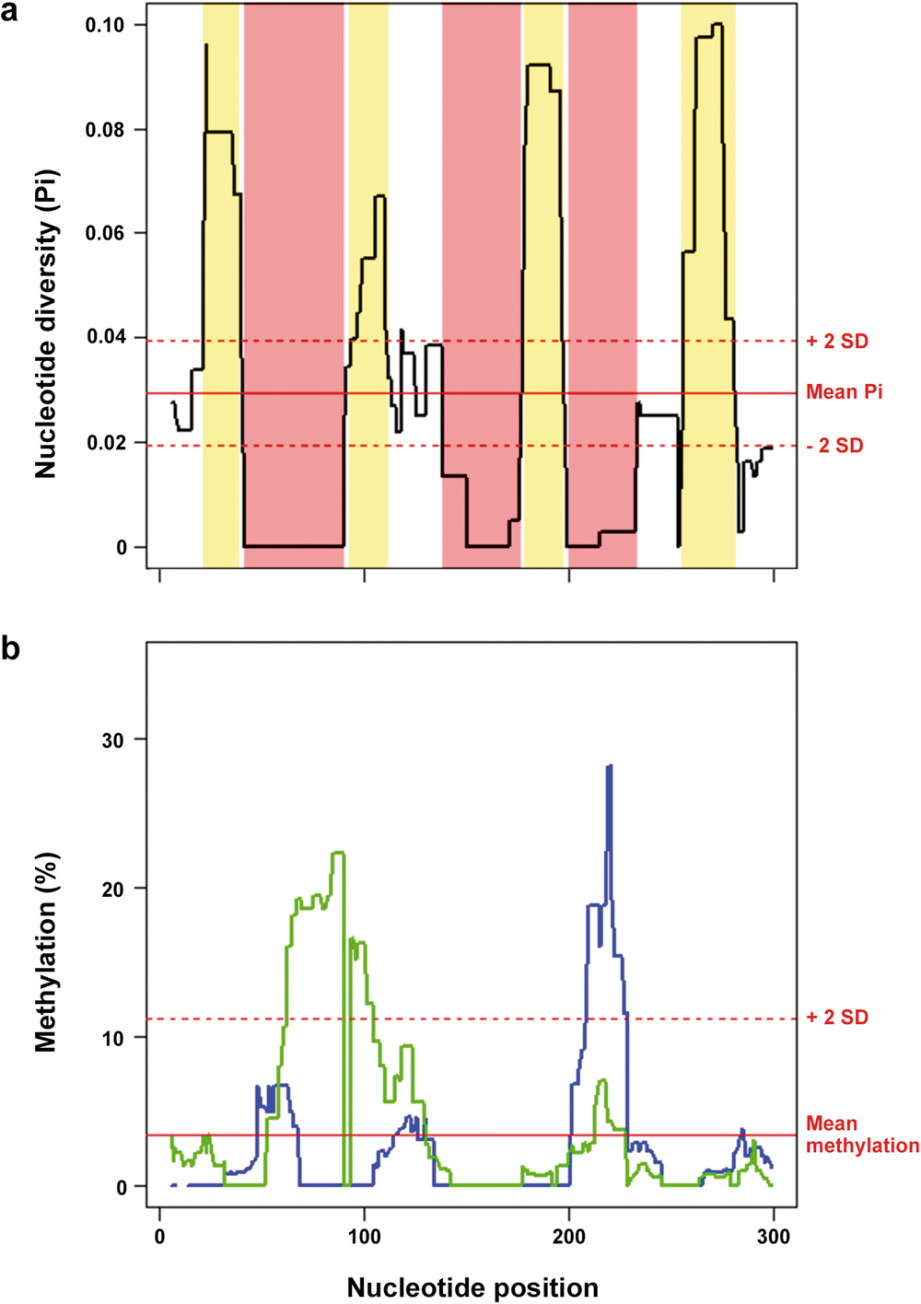
Nucleotide diversity and cytosine methylation throughout SSUsat monomer sequence. Sliding window analysis of the distribution of the nucleotide diversity (**a**), computed using a 10 bp overlapping sliding window, along the SSUsat sequence showing conserved (pinkish) and variable (yellowish) regions; the red lines indicate mean (±2 SD) diversity (Pi). The same type of analysis applied to the proportion of methylated cytosines (**b**), also computed using a 10 bp overlapping sliding window, for direct (green line) and complementary (blue line) strands of the SSUsat; red lines indicate mean (+2 SD) methylation values.

The 71 SSUsat sequences, coming from *S. subtruncata*, *S. solida*, and *M. stultorum*, were aligned and compared (Supplementary Fig. 2). Among them, 66 sequences were 315 bp long, one showing a single nucleotide insertion and five having a single nucleotide deletion. The overall interspecific nucleotide diversity was low (Pi = 0.0290) and comparable to the intraspecific diversities (*S. subtruncata*: 0.0294, *S. solida*: 0.0340, *M. stultorum*: 0.0290), thus denoting a high degree of sequence similarity within and among species. Phylogenetic analysis of the SSUsat sequences clearly revealed clustering of monomer variants from different taxa, indicating a lack of correlation between monomer variants and a given taxon (Fig. 4). Regarding nucleotide diversity, its distribution along the satellite sequence was not homogeneous; variable positions were mostly piled up at four 15 bp long regions alternating with three highly conserved regions (Fig. 5a).

### Chromosomal localization of the SSUsat DNA

**Figure 6:**
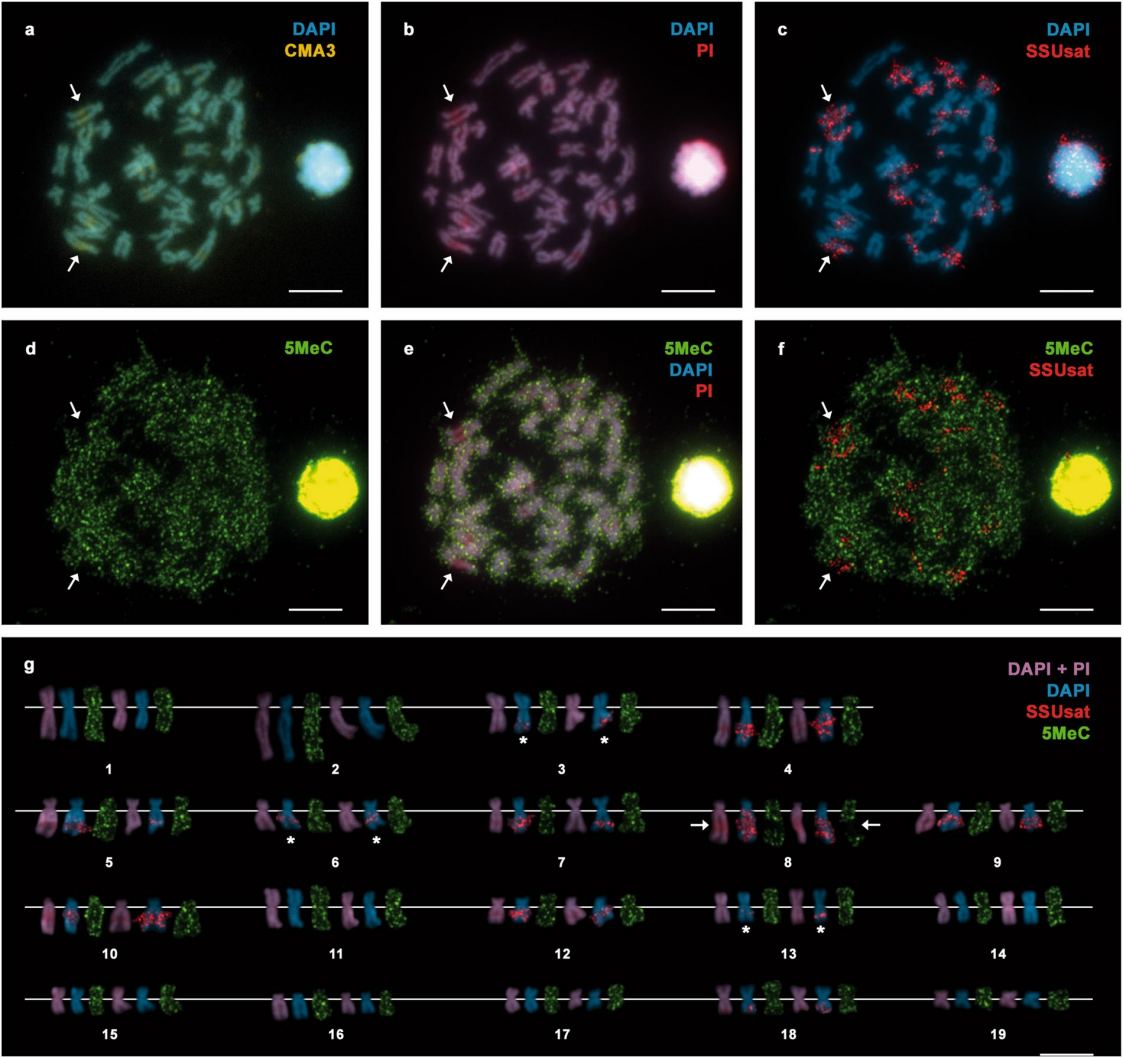
Chromosomal location of G + C-rich bands, SSUsat and methylated regions in *Spisula subtruncata*. DAPI/CMA (**a**) followed by DAPI/PI staining (**b**) and FISH using the SSUsat probe (**c**) on the same metaphase plate and the corresponding karyotype (**g**) show that SSUsat signals mainly co-locate with the CMA positive, DAPI negative, G + C-rich intercalary heterochromatic regions on chromosome pairs 4, 5, 7, 8, 9, 10 and 12 and with the subterminal G + C-rich heterochromatin associated with the NOR on chromosome pair 18. Additional intercalary SSUsat signals are also present in regions not showing conspicuous CMA + /DAPI- staining in the long arms of chromosome pairs 3, 6 and 13 (asterisks in **g**). Immunolocalization of 5-methylcytosine (5-MeC) in the same metaphase plate (**d**–**f**) and the corresponding karyotype (**g**) mainly shows a homogeneous distribution of the 5-MeC signals with two main exceptions; the absolute lack of methylation signals on the biggest, intercalary CMA + /DAPI-, G + C-rich, heterochromatic, SSUsat-bearing band on the long arm of chromosome 8 (arrows in **a**–**g**) and the strong methylation showed by the subterminal NOR in one of the homologous chromosomes of pair 18. Scale bars, 5 μm.

The chromosomal location of the SSUsat was determined by FISH on mitotic metaphase plates previously stained with DAPI/CMA and DAPI/PI to evidence G + C-rich chromatin regions (Fig. 6a,b). As previously shown^42^, the karyotype of *S. subtruncata* is composed of 2n = 38 chromosomes and presents CMA positive/DAPI negative, intercalary G + C-rich bands on the long arms of chromosome pairs 4, 5, 7, 8, 9, 10, and 12, and a subterminal band, coincident with the NOR, on the long arm of pair 18 (Fig. 6g). As determined by barium hydroxide C-banding, these G + C-rich bands were heterochromatic^42^. Although most of the FISH signals obtained with the SSUsat probes overlapped the heterochromatic regions situated in those chromosome pairs (Fig. 6c,g), additional SSUsat signals, apparently situated outside heterochromatic regions, were detected on the long arms of the chromosome pairs 3, 6, and 13 (asterisks in Fig. 6g). Whilst on the other eight chromosome pairs (1, 2, 11, 14, 15, 16, 17, and 19) SSUsat signals were not detected.

FISH using the SSUsat probe on *S. solida* and *M. stultorum* chromosome spreads revealed inconclusive results, possibly resultant of a low number of copies and/or the absence of long (>1.5 kb) arrays of SSUsat monomers in these species.

### Cytosine methylation in the genome of *S. subtruncata*

The distribution of methylated regions on the chromosomes of *S. subtruncata* was analyzed by immunostaining employing an antibody against 5-MeC. The overall fluorescence was rather low and image acquisition required long exposure times. As shown in Fig. 6d–f, the 5-MeC signals were homogeneously distributed along most regions of the *S. subtruncata* chromosomes (Fig. 6d,g). In contrast, the most prominent G + C-rich heterochromatic band, which presented the strongest SSUsat FISH signals, did not display any fluorescence (chromosome 8, arrows in Fig. 6). Although these results clearly show a low degree of methylation of some of the G + C-rich heterochromatic regions, this methodology does not allow for a quantitative measurement of the global genome methylation. Therefore other approaches were employed to better assess the methylation degree of the cut trough shell genome.

The detection of possible differences in the degree of cytosine methylation among *S. subtruncata* adult specimens was determined through a MSAP experiment. Comparisons of banding patterns obtained after *Eco*RI + *Hpa*II and *Eco*RI + *Msp*I digestions demonstrated that out of a total of 288 methylation-susceptible loci, 103 (36.79%) were polymorphic. Surprisingly, no significant intraindividual differences were found between *Hpa*II and *Msp*I restriction banding profiles (AMOVA; Φ_*PT*_ = 0.0000, p = 0.513). Furthermore, the principal cooordinate analysis of the MSAP data showed that the *Hpa*II and *Msp*I profiles from a single specimen are quite close, without any other clustering trend (Supplementary Fig. 4). These results strongly indicate that the level of cytosine methylation in adult cut trough shell genome is very low and were further corroborated by data obtained by ELISA immunoassays which demonstrated that less than 1.3% of the genome (1.13 ± 0.22) was methylated in *S. subtruncata*.

In a further attempt to establish the degree of methylation of the SSUsat sequences, we treated genomic DNA with bisulfite and sequenced the bisulfite modified monomers. To avoid biases associated with spontaneous point mutations from cytosine to thymine in the native DNA, methylation data was normalized by dividing the proportion of cytosine after bisulfite transformation by the proportion of thymine and cytosine in the initial monomer pool. The efficiency of the bisulfite conversion, checked by treating PCR amplified monomers in parallel with genomic DNA samples, was 98.7%. The results obtained after analysing a total of 33 bisulfite transformed fragments recovered for the direct strand and 26 for the complementary, outlined in Table 1 and Supplementary Fig. 5, indicate that the 3.38% of the cytosine sites in the SSUsat are methylated, three times the genomic mean, without main differences between strands (3.75% and 3.00%) and that the proportion of methylated cytosine was higher at CpG sites (5.65%) than at CHH (2.69%) and CHG (1.79%) ones. Regarding the methylation status of the three CCGG restriction targets for *Hpa*II/*Msp*I, most of them were unmethylated and two showed internal cytosine hemimethylation, therefore being cut by the two isoschizomers. On the other hand, our results also indicated that methylation is unevenly distributed along the satellite (Fig. 5b) and negatively correlated with nucleotide diversity both for the direct (ρ = −0.1340, p = 0.0213) and the complementary (ρ = −0.2505, p = 0.0000) strands and also for both strands taken together (ρ = −0.2975, p = 0.0000). Furthermore, the highest methylated regions (nucleotide positions 60–89 and 209–221) coincide with two of the three most conserved domains (nucleotide positions 41–89 and 199–232) in the monomer sequence.

**Table 1:** Proportion (%) of methylated cytosines in SSUsat monomers according C site type and DNA strand.

Site	Strand		
	Direct	Complementary	Both
CG	6.18	5.11	5.65
CHG	2.97	0.61	1.79
CHH	2.11	3.28	2.69
All C	3.75	3.00	3.38

## Discussion

SatDNA sequences have been found in many bivalve mollusc species. These organisms have been considered a good model for the study of satDNA dynamics, with satellite families ubiquitous at higher taxonomic levels^19, 20^, and fast evolving ones, restricted to a limited number of congeneric species^16, 18^. The SSUsat described here is the first satellite DNA sequence found in trough shell genomes and, as BLAST searches did not reveal significant similarity with any other DNA sequence stored in GenBank, representing a new satellite family. SSUsat content in *S. subtruncata*, making at least 1.3% of the genome, was among the highest detected until now for a homogeneous satDNA family in bivalves, being comparable with the abundance of the DTF2 satellite of the wedge shell *D. trunculus* ^30, 44^ or with the BIV160 satDNA of *R. decussatus*, which represents 2% of its genome^19^. The different amplification and distribution of the SSUsat in the genomes of three trough shell species, abundant and presumably organized into long arrays in *S. subtruncata* and low copy in *S. solida* and *M. stultorum*, but absent in the more distant *D. trunculus*, is concordant with the library model of satellite DNA evolution^10, 22^.

Although initially thought to be junk DNA, an accumulating amount of evidence from the last few years has suggested that satellite DNAs are involved in heterochromatin dynamics, centromere function and epigenetic silencing^6, 45, 46^. The irregular distribution of nucleotide diversity along satellite monomers is considered an indication of the evolutionary constraints imposed on segments of monomer sequences, whilst more variable segments would correspond to regions of a lesser relevance^47, 48^. The non-randomness of the nucleotide diversity in the SSUsat, showing three highly conserved domains interspersed with four variable regions, constitute an additional example of that paradigm.

As in many other invertebrates, G + C contents of bivalve genomes are relatively low: 31.65% in the mussel *Mytilus galloprovincialis* ^3^, 33.69% in the pearl oyster *Pinctada imbricata fucata* ^1^, 35.30% in the freshwater clam *Corbicula fluminea* (https://www.ncbi.nlm.nih.gov/genome/15808), and 36.60% in the oyster *Crassostrea gigas* ^2^. Heterochromatin content is also scarce in bivalves and only the wedge shell *D. trunculus* ^30, 39^, the zebra mussel *Dreissena polymorpha* ^40, 41^ and the trough shells *S. subtruncata* and *M.stultorum* ^42^ characteristically present relatively high amounts of C-banded heterochromatin. Differential staining with base-specific fluorochromes demonstrated that these heterochromatic bands were G + C-rich, i.e. showed higher G + C content than the surrounding euchromatin^30, 42^. As the SSUsat is the main component of the heterochromatic bands in *S. subtruncata* and its G + C-richness is around 44%, it can be inferred that the overall G + C-content of *S. subtruncata* must be below that value as it is in other bivalves.

Although DNA methylation has been traditionally interpreted as an epigenetic mark in the eukaryotic genome, regulating both gene transcription and heterochromatin formation and inactivating transposition^24^, most cytosine methylation data was obtained from a few vertebrate and plant genomes and even less from invertebrates^25, 49^. DNA methylation patterns in invertebrates differs from those found in vertebrates, being generally characterized by low methylation levels^25, 28^; this is also the case in bivalves^25, 32, 49^. In concordance, the level of cytosine methylation of the genome for *S. subtruncata* is very low (1.3%) but showing about thrice that value for the SSUsat. Increased methylation levels have also been reported for satellite DNAs in other invertebrates such as the red flour beetle *Tribolium castaneum* ^50^, the wedge shell *D. trunculus* ^30^, and the oyster *C. gigas* ^29^. In *T. castaneum* the satDNA methylation is twice the genome level in adults^50^ whereas in *C. gigas* is similar to the genome level but twice as high as intergenic regions with no annotations^29^. This situation may be related to the essential role played by DNA methylation in regulating and maintaining heterochromatin^51^ as its changes in many cases parallel those in histone modifications^51^.

Interestingly, SSUsat monomer sequence is highly conserved among Mactridae species and methylation along the SSUsat monomer is inversely correlated with nucleotide diversity, being the two highest methylated regions in SSUsat coincident with two of the three most conserved sequence segments. This is also in agreement with data from the oyster *C. gigas* in which DNA methylation is not randomly distributed but preferentially affects the most conserved repetitive sequences^29^. As evolutionary constraints are predicted for the most conserved sequences on satellite DNAs^47, 48^, the distribution of DNA methylation on SSUsat also points to an important functional role of these modifications.

Moreover, SSUsat methylation affects not only to the cytosine on CpG dinucleotides but also, though in a lesser extent, to CHH and CHG trinucleotides; non-CpG methylation is an abundant phenomenon playing structural and repressive roles in plants^23^ but scarce and without clear relevance in animals^29^. Nevertheless, relatively high levels of non-CpG methylation have been reported for some animal species^29, 50, 52^ and some studies correlate this kind of methylation with reduced gene expression and inactivation of distal regulatory elements in stem cells^53^ and neurons^54, 55^ in mammals.

On the other hand, the clear absence of methylation signals on the heterochromatic regions of chromosome pair 8 could indicate that methylated and non-methylated SSUsat monomers are not randomly distributed along heterochromatin. Somewhat similar results were previously found for the satellite family pEV in the wild beet *Beta procumbens* in which, as demonstrated by immunostaining with an antibody against 5-MeC, the methylation of the heterochromatic regions bearing the pEV satellite showed a certain degree of variation^23^. Whether this is a consequence of particular life stage- or tissue-specific methylation/demethylation processes or any other cause needs to be evaluated.

## Materials and Methods

### Biological samples and DNA isolation

Adult specimens of the cut trough shell *Spisula subtruncata* (da Costa, 1778), the thick trough shell *Spisula solida* (Linnaeus, 1758), the rayed trough shell *Mactra stultorum* (Linnaeus, 1758) and the wedge shell *Donax trunculus* (Linnaeus, 1758) were collected from natural populations in Galicia (NW Spain). At least five individuals per species were analyzed.

Genomic DNA was isolated from adductor muscles using the E.Z.N.A. ® Mollusc DNA Kit (OMEGA). *S. subtruncata* genomic DNA was subjected to partial digestion with *Hae*III (New England Biolabs), and the products were separated by agarose gel electrophoresis producing a ladder-like profile. Fragments up to tetramer were recovered from the agarose gels using the QIAquick Gel Extraction Kit (Qiagen).

### Southern and dot blot hybridisation

Genomic DNAs were digested with *Hae*III (Fermentas), *Hpa*II (Fermentas), *Msp*I (Promega), *Pvu*II (New England Biolabs) and *Taq*I (Roche). Restriction products were resolved by gel electrophoresis, transferred to positively charged nylon membranes (Roche), and hybridized overnight with the DIG-labelled SSUsat probe. To test homology in low, moderate and high stringency conditions, different experimental temperatures (60, 65 and 68 °C) were used. Signals were detected with alkaline phosphatase conjugated anti-digoxigenin antibodies (Roche) using CDP-Star (Roche) as substrate.

The proportions of the satellite present in the genomes of *S. subtruncata*, *S. solida*, *M. stultorum* and *D. trunculus* were estimated by dot blotting. Serial dilutions of genomic DNAs (10–250 ng) and cloned SSUsat (0.3–10 ng) were dot-blotted onto positively charged nylon membranes (Roche) and hybridized with SSUsat probes. Hybridisation signals were quantified using ImageJ (http://imagej.nih.gov/ij/).

### Cloning, sequencing, and sequence analysis

DNA fragments were cloned into XL10-Gold Ultracompetent Cells (Agilent Technologies) using a *Sma*I-digested pUC19 plasmid vector. Positive clones were purified using the High Pure Plasmid Isolation Kit (Roche) and commercially sequenced (Macrogen Inc., Amsterdam, the Netherlands) using M13/pUC forward and reverse primers. The resulting sequences were aligned using ClusterW via MEGA V7^56^ applying default parameters.

On the basis of the *S. subtruncata* aligned sequences (SSUsat sequences in Supplementary Fig. 2) and using the Lasergene PrimerSelect software (Dnastar), we designed a set of divergent primers (SSUsatPR1: 5′-AACCGCGGTGCAAGTTCG-3′; SSUsatPR2: 5′-TAACCTGTTTTCGCGATGTTTACG-3′) in order to explore the conservation of the sequences in closely (*S. solida*, *M. stultorum*) and distantly (*D. trunculus*) related species. PCR primers were designed within the most conserved regions of *S. subtruncata* SSUsat sequences (Supplementary Fig. 2). PCR conditions included an initial denaturation at 94 °C for 3 min and 35 cycles of 94 °C for 20 s, 63 °C for 20 s and 72 °C for 1 min. A final extension at 72 °C for 7 min was applied. All reactions were performed in an Applied Biosystems 2720 Thermal Cycler. PCR products (comprising a full-size monomer plus flanking regions) were cloned into XL10-Gold Ultracompetent Cells (Agilent Technologies) using the pGEM®-T Easy Vector System (Promega). Positive clones were colony-PCR amplified with the vector specific primers (M13F, M13R-40) and sequenced as described above. The sequences were aligned using ClusterW via MEGA V7^56^ applying default parameters. Phylogenetic analysis was carried out with the Maximum Likelihood method based on the Kimura 2-parameter model plus Gamma implemented in MEGA V7^56^. The distribution of the nucleotide diversity (Pi) was estimated using DnaSP v5^57^ as described in Plohl *et al*.^19^. Sequence segments were considered conserved or variable only if Pi differed from the mean value in more than two times the standard deviation.

The SSU consensus sequence was subjected to BLAST analyses^58^ using different BLAST algorithms: MegaBLAST (revealing highly similar sequences), discontiguous MegaBLAST (disclosing more dissimilar sequences), and BLASTN (detecting somewhat similar sequences). The SSUsat consensus sequence was also screened against RepBase, a database of representative repetitive sequences from eukaryotic species, using CENSOR software^59^.

Local blast searches against *Crassostrea gigas* genome and partial genomic libraries of other bivalve species (*Donax trunculus, Ruditapes decussates*, and *Ruditapes philippinarum*) was performed with Geneious 9.1.7 software (Biomatters Ltd.). *C. gigas* genome used for this purpose was WGS sequence set AFTI01000000. Partial genomic libraries of other bivalves included datasets with the following accession numbers: KC981676 - KC981759, KU682284 - KU682293, KU682294 - KU682299, KU682300 - KU682313, KU682314 - KU682355, KY400497 - KY400517, EU275729 - EU275748, EU925654 - EU925762, AY940458 - AY940475, X94535 - X94546, X86930 - X86936, together with yet unpublished nucleotide sequences enriched in repetitive DNAs.

### Immunodetection and fluorescent *in situ* hybridisation

Mitotic chromosomes were obtained as indicated in García-Souto *et al*.^38, 42^. Specimens were treated with colchicine (0.005%, 8 h) before dissection. Gills were hypotonised in diluted seawater and fixed with ethanol-acetic acid (3:1). Gill fragments were immersed in acetic acid (60%) and the resulting cell suspension dropped onto glass slides.

Immunodetection of 5-methylcytosine (5-MeC) was performed with mouse anti-5-MeC (Eurogentec) following supplier indications as modified by Covelo-Soto *et al*.^60^. After detection with fluorescein isothiocyanate (FITC) conjugated goat anti-mouse antibodies (Sigma), chromosomes were counterstained with CMA (0.25 mg/mL) and DAPI (0.14 μg/mL in 2x SSC). Chromosome slides were mounted with antifade (Vectashield, Vector). After microscopy and image acquisition, chromosome preparations were re-stained with DAPI and propidium iodide (PI: 0.07 μg/mL in 2x SSC) and photographed again.

Fluorescent *in situ* hybridisation (FISH) with digoxigenin-labelled SSUsat probes was performed as described previously^38, 42, 59, 61^. Chromosome spreads were treated with RNase and pepsin before denaturation in 70% formamide (70 °C, 2 min). After overnight hybridisation and stringency washes (50% formamide and 1xSSC, 45 °C), signal detection was accomplished with mouse anti-digoxigenin, goat anti-mouse tetramethylrhodamine isothiocyanate (TRITC) and rabbit anti-goat TRITC antibodies (Sigma). Slides were then counterstained with DAPI and mounted with antifade.

Chromosome analysis and imaging was performed with a Nikon Eclipse E800 microscope equipped with an epifluorescence system and a DS-Qi1Mc CCD camera controlled by NIS-Elements (Nikon). At least 20 complete metaphase plates from five individuals of each species were examined. Processing of the images was performed with Adobe Photoshop.

### Bisulfite sequencing

Genomic DNA of *S. subtruncata* was converted with sodium bisulfite, using the EZ DNA Methylation™ Kit (ZYMO Research) as recommended by the manufacturer. After transformation, entire SSUsat monomers were recovered from both strands using four sets of primers, two of them targeting the direct strand (SSUsat1MD F/R: 5′-AACCGCGGTGYAAGTTYGA-3′, 5′-ATAACCTRTTTTCRCRATRTTTAC-3′; SSUsat2MD F/R: 5′-CCGGAAGTTACACATTTYGGA-3′, 5′CAAAAAATGRCCTCCAACTTRCA-3′) and the other two the complementary strand (SSUsat1MC F/R: 5′-CAAAAAATGGGYTYGAAYTTGYA-3′, 5′-CCGGAARTTACACATTTCRRA-3′; SSUsat2MC F/R: 5′-ATAACYTGTTTTYGYGATGTTTAY-3′, 5′AACCGCGRTRCAARTTCR A-3′). Following an initial denaturation at 95 °C (5 min), 35 amplification cycles of 95 °C (15 s), 52 °C (15 s) and 72 °C (15 s) and a final extension step at 72 °C (7 min) were applied. Amplicons were cloned using the NZY-A PCR cloning kit (NZYTECH) and sequenced using the kit vector primers. A total of 33 bisulfite transformed fragments (15 external and 18 internal) recovered for the direct strand, and 26 (15 external and 11 internal) recovered for the complementary strand were aligned with the untransformed SSUsat library using MEGA V7^56^. All conserved cytosine positions were manually screened for methylation events. Sequences corresponding to the primers were excluded from the analysis.

The relationship between methylation level and sequence conservation was assessed by means of the Spearman correlation coefficient.

### ELISA and Methylation-Sensitive Amplified Polymorphism

The overall 5-MeC content in the genome of S. *subtruncata* was determined using a monoclonal antibody specific for 5-MeC (5-MeC ELISA Kit, ZYMO Research) following the manufacturer’s instructions. The results were expressed as the proportion of 5-MeC in the DNA sample. The standard curve was generated using the controls included in the kit.

A methylation sensitive amplified polymorphism (MSAP) assay^62, 63^ was performed on the same samples. This assay is based on the differential cleavage reactivity of the isoschizomers *Hpa*II and *Msp*I to the cytosine methylation of their target and permits the assessment of differences in methylation patterns within and among specimens. After *Eco*RI + *Hpa*II and *Eco*RI + *Msp*I digestion of genomic DNA from eight specimens of *S. subtruncata*, the restriction profiles obtained by capillary electrophoresis (ABI Prism 310 Genetic Analyzer, Applied Biosystems) using a 500 ROX size standard (GeneScan) were scored with GeneMapper v.3.7 (Applied Biosystems) and compared using the R package MSAP^64^ and GeneAlEx 6.502^65^.

### Data availability

All data generated or analysed during this study are included in this published article (and its Supplementary Information files)

## Electronic supplementary material

Supplementary Information

## References

[1] Takeuchi T (2012). Draft genome of the pearl oyster *Pinctada fucata*: a platform for understanding bivalve biology. DNA Res..

[2] Zhang G (2012). The oyster genome reveals stress adaptation and complexity of shell formation. Nature.

[3] Murgarella M (2016). A first insight into the genome of the filter-feeder mussel *Mytilus galloprovincialis*. PLoS One.

[4] Jurka J, Kapitonov VV, Kohany O, Jurka MV (2007). Repetitive sequences in complex genomes: structure and evolution. Annu. Rev. Genomics Hum. Genet..

[5] Plohl M, Luchetti A, Meštrović N, Mantovani B (2008). Satellite DNAs between selfishness and functionality: structure, genomics and evolution of tandem repeats in centromeric (hetero) chromatin. Gene.

[6] Plohl M (2010). Those mysterious sequences of satellite DNAs. Period. Biol..

[7] Novák P, Neumann P, Pech J, Steinhaisl J, Macas J (2013). RepeatExplorer: a Galaxy-based web server for genome-wide characterization of eukaryotic repetitive elements from next-generation sequence reads. Bioinformatics.

[8] Ruiz-Ruano FJ, López-León MD, Cabrero J, Camacho JPM (2016). High-throughput analysis of the satellitome illuminates satellite DNA evolution. Sci. Rep..

[9] Heckmann S (2013). The holocentric species *Luzula elegans* shows interplay between centromere and large-scale genome organization. Plant J..

[10] Fry K, Salser W (1977). Nucleotide sequences of HS-alpha satellite DNA from kangaroo rat *Dipodomys ordii* and characterization of similar sequences in other rodents. Cell.

[11] Meštrović N, Plohl M, Mravinac B, Ugarković Ð (1998). Evolution of satellite DNAs from the genus *Palorus* - experimental evidence for the “library” hypothesis. Mol. Biol. Evol..

[12] Passamonti M, Mantovani B, Scali V (1998). Characterization of a highly repeated DNA family in Tapetinae species (Mollusca Bivalvia: Veneridae). Zool. Sci..

[13] Canapa A, Barucca M, Cerioni PN, Olmo E (2000). A satellite DNA containing CENP-B box-like motifs is present in the Antarctic scallop *Adamussium colbecki*. Gene.

[14] Martínez-Lage A (2002). Comparative analysis of different satellite DNAs in four *Mytilus* species. Genome.

[15] López-Flores I (2004). The molecular phylogeny of oysters based on a satellite DNA related to transposons. Gene.

[16] Martínez-Lage A, Rodríguez-Fariña F, González-Tizón A, Méndez J (2005). Origin and evolution of *Mytilus* mussel satellite DNAs. Genome.

[17] Biscotti MA (2007). Repetitive DNA, molecular cytogenetics and genome organization in the king scallop (*Pecten maximus*). Gene.

[18] López-Flores I (2010). Molecular characterization and evolution of an interspersed repetitive DNA family of oysters. Genetica.

[19] Plohl M (2010). Long-term conservation vs high sequence divergence: the case of an extraordinarily old satellite DNA in bivalve mollusks. Heredity.

[20] Petraccioli A (2015). A novel satellite DNA isolated in *Pecten jacobaeus* shows high sequence similarity among molluscs. Mol. Genet. Genomics.

[21] Ugarković Đ (2005). Functional elements residing within satellite DNAs. EMBO Rep..

[22] Plohl, M., Meštrović, N. & Mravinac, B. Satellite DNA evolution in *Repetitive DNA. Genome Dynamics 7* (ed. Garrido-Ramos, M. A.) 126-152 (Karger, 2012). 10.1159/000337122 22759817

[23] Schmidt M (2014). Cytosine methylation of an ancient satellite family in the wild beet *Beta procumbens*. Cytogenet. Genome Res..

[24] Bird A (2002). DNA methylation patterns and epigenetic memory. Genes Dev..

[25] Gavery MR, Roberts SB (2010). DNA methylation patterns provide insight into epigenetic regulation in the Pacific oyster (*Crassostrea gigas*). BMC Genomics.

[26] Gavery MR, Roberts SB (2013). Predominant intragenic methylation is associated with gene expression characteristics in a bivalve mollusc. PeerJ..

[27] Glastad KM, Hunt BG, Yi SV, Goodisman MAD (2011). DNA methylation in insects: on the brink of the epigenomic era. Insect Mol. Biol..

[28] Keller TE, Han P, Yi SV (2016). Evolutionary transition of promoter and gene body DNA methylation across Invertebrate -Vertebrate boundary. Mol. Biol. Evol..

[29] Wang X (2014). Genome-wide and single-base resolution DNA methylomes of the Pacific oyster *Crassostrea gigas* provide insight into the evolution of invertebrate CpG methylation. BMC Genomics.

[30] Petrović V (2009). A GC-rich satellite DNA and karyology of the bivalve mollusk *Donax trunculus*: a dominance of GC-rich heterochromatin. Cytogenet. Genome Res..

[31] Šatović E, Plohl M (2013). Tandem repeat-containing MITEs in the clam *Donax trunculus*. Genome Biol. Evol..

[32] Regev A, Lamb MJ, Jablonka E (1998). The role of DNA methylation in invertebrates: developmental regulation or genome defense?. Mol. Biol. Evol..

[33] Leitão, A. & Chaves, R. Banding for chromosomal identification in bivalves. A 20-year history In *Dynamic Biochemistry, Process Biotechnology and Molecular Biology 2 (Special* Issue *1)* (ed. Russo, R.) 44-49 (Global Science Books, 2008).

[34] Pérez-García C, Cambeiro JM, Morán P, Pasantes JJ (2010). Chromosomal mapping of rDNAs, core histone genes and telomeric sequences in *Perumytilus purpuratus* (Bivalvia: Mytilidae). J. Exp. Mar. Biol. Ecol..

[35] Pérez-García C, Guerra-Varela J, Morán P, Pasantes JJ (2010). Chromosomal mapping of rRNA genes, core histone genes and telomeric sequences in *Brachidontes puniceus* and *Brachidontes rodriguezi* (Bivalvia: Mytilidae). BMC Genet..

[36] Pérez-García C, Morán P, Pasantes JJ (2011). Cytogenetic characterization of the invasive mussel species *Xenostrobus securis* Lmk. (Bivalvia: Mytilidae). Genome.

[37] Carrilho J, Pérez-García C, Leitão A, Malheiro I, Pasantes JJ (2011). Cytogenetic characterization and mapping of rDNAs, core histone genes and telomeric sequences in *Venerupis aurea* and *Tapes rhomboides* (Bivalvia: Veneridae). Genetica.

[38] García-Souto D, Pérez-García C, Morán P, Pasantes JJ (2015). Divergent evolutionary behavior of H3 histone gene and rDNA clusters in venerid clams. Mol. Cytogenet..

[39] Martínez A, Marinas L, González-Tizón A, Méndez J (2002). Cytogenetic characterization of *Donax trunculus* (Bivalvia: Donacidae) by means of karyotyping, fluorochrome banding and fluorescent *in situ* hybridisation. J. Mollusc. Stu..

[40] Woznicki P, Boron A (2003). Banding chromosome patterns of zebra mussel *Dreissena polymorpha* (Pallas) from the heated Konin lakes system (Poland). Caryologia.

[41] Boron A, Woznicki P, Skuza L, Zielinski R (2004). Cytogenetic characterization of the zebra mussel *Dreissena polymorpha* (Pallas) from Miedwie Lake, Poland. Folia Biol. (Kraków).

[42] García-Souto D, Pérez-García C, Kendall J, Pasantes JJ (2016). Molecular cytogenetics in trough shells (Mactridae, Bivalvia): Divergent GC-rich heterochromatin content. Genes.

[43] Bao W, Kojima KK, Kohany O (2015). Repbase Update, a database of repetitive elements in eukaryotic genomes. Mob. DNA.

[44] Petrović V, Plohl M (2005). Sequence divergence and conservation in organizationally distinct subfamilies of *Donax trunculus* satellite DNA. Gene.

[45] Zhimulev IF, Belyaeva ES (2003). Intercalary heterochromatin and genetic silencing. BioEssays.

[46] Ugarković Đ (2008). Satellite DNA libraries and centromere evolution. Open Evol. J..

[47] Mravinac B, Plohl M, Ugarković Ð (2005). Preservation and high sequence conservation of satellite DNAs suggest functional constraints. J. Mol. Evol..

[48] Meštrović N (2013). Conserved DNA motifs, including the CENP-B Box-like, are possible promoters of satellite DNA array rearrangements in Nematodes. PLoS One.

[49] Rivière G (2014). Epigenetic features in the oyster *Crassostrea gigas* suggestive of functionally relevant promoter DNA methylation in invertebrates. Front. Physiol..

[50] Song, X. *et al*. Genome-wide DNA methylomes from discrete developmental stages reveal the predominance of non-CpGmethylation in *Tribolium castaneum*. DNA Res. **24**, doi:10.1093/dnares/dsx016 (2017). 10.1093/dnares/dsx016 PMC5737696 28449092

[51] Feng W, Michaels SD (2015). Accessing the inaccessible: The organization, transcription, replication, and repair of heterochromatin in plants. Annu. Rev. Genet..

[52] Su Z, Han L, Zhao Z (2011). Conservation and divergence of DNA methylation in eukaryotes. New insights from single base-resolution DNA methylomes. Epigenetics.

[53] Lister R (2009). Human DNA methylomes at base resolution show widespread epigenomic differences. Nature.

[54] Guo JU (2014). Distribution, recognition and regulation of non-CpG methylation in the adult mammalian brain. Nat. Neurosci..

[55] Keown CL (2017). Allele-specific non-CG DNA methylation marks domains of active chromatin in female mouse brain. Proc. Natl. Acad. Sci. USA.

[56] Kumar S, Stecher G, Tamura K (2016). MEGA7: Molecular Evolutionary Genetics Analysis version 7.0 for bigger datasets. Mol. Biol. Evol..

[57] Librado P, Rozas J (2009). DnaSP v5: A software for comprehensive analysis of DNA polymorphism data. Bioinformatics.

[58] Altschul SF, Gish W, Miller W, Myers EW, Lipman DJ (1990). Basic local alignment search tool. J. Mol. Biol..

[59] Kohany O, Gentles AJ, Hankus L, Jurka J (2006). Annotation, submission and screening of repetitive elements in Repbase: RepbaseSubmitter and Censor. BMC Bioinformatics.

[60] Covelo-Soto L, Morán P, Pasantes JJ, Pérez-García C (2014). Cytogenetic evidences of genome rearrangement and differential epigenetic chromatin modification in the sea lamprey (*Petromyzon marinus*). Genetica.

[61] Pérez-García C, Morán P, Pasantes JJ (2014). Karyotypic diversification in *Mytilus* mussels (Bivalvia: Mytilidae) inferred from chromosomal mapping of rRNA and histone gene clusters. BMC Genet..

[62] Díaz-Freije E, Gestal C, Castellanos-Martínez S, Morán P (2014). The role of DNA methylation on *Octopus vulgaris* development and their perspectives. Front. Physiol..

[63] Ardura A, Zaiko A, Morán P, Planes S, García-Vázquez E (2017). Epigenetic signatures of invasive status in populations of marine invertebrates. Sci. Rep..

[64] Pérez‐Figueroa A (2013). MSAP: a tool for the statistical analysis of methylation‐sensitive amplified polymorphism data. Mol. Ecol. Resour..

[65] Peakal R, Smouse PE (2012). GenAlEx 6.5: genetic analysis in Excel. Population genetic software for teaching and research-an update. Bioinformatics.

