# Supplementary material for: Methylation profile of a satellite DNA constituting the intercalary G+C-rich heterochromatin of the cut trough shell Spisula subtruncata (Bivalvia, Mactridae)
Source: Sci Rep. 2017 Jul 31;7:6930. doi: 10.1038/s41598-017-07231-7 (PMC5537241; doi:10.1038/s41598-017-07231-7)
Supplement: Supplementary file 1 — Supplementary Information [file 41598_2017_7231_MOESM1_ESM.pdf]

Methylation profile of a satellite DNA constituting the intercalary G+C-rich heterochromatin of the cut trough shell *Spisula subtruncata* (Bivalvia, Mactridae)

Daniel García-Souto, Brankica Mravinac, Eva Šatović, Miroslav Plohl,

Paloma Morán, Juan J. Pasantes

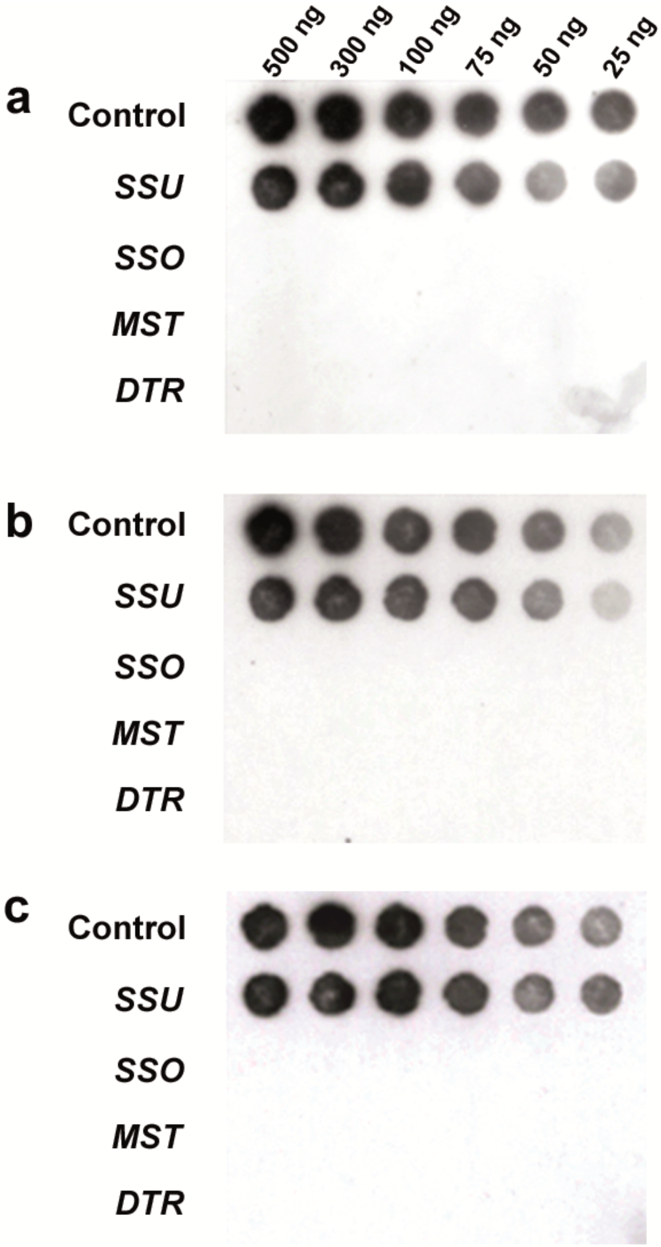

Supplementary Figure 1. Quantification of SSUsat by dot blot hybridisation. Quantification analysis of SSUsat by dot blot hybridisation at high (68 ºC) (**a**), medium (65 ºC) (**b**) and low (60 ºC) (**c**) stringency on *Spisula subtruncata* (SSU), *Spisula solida* (SSO), *Mactra stultorum* (MST) and *Donax trunculus* (DTR) genomic DNA. Serial dilutions of genomic DNAs from SSU, SSO, MST and DTR and a control of cloned satellites were dot blotted and denatured onto nitrocellulose membranes and hybridised with an SSUsat monomer probe. The amounts of dot blotted DNA (ng) are indicated.

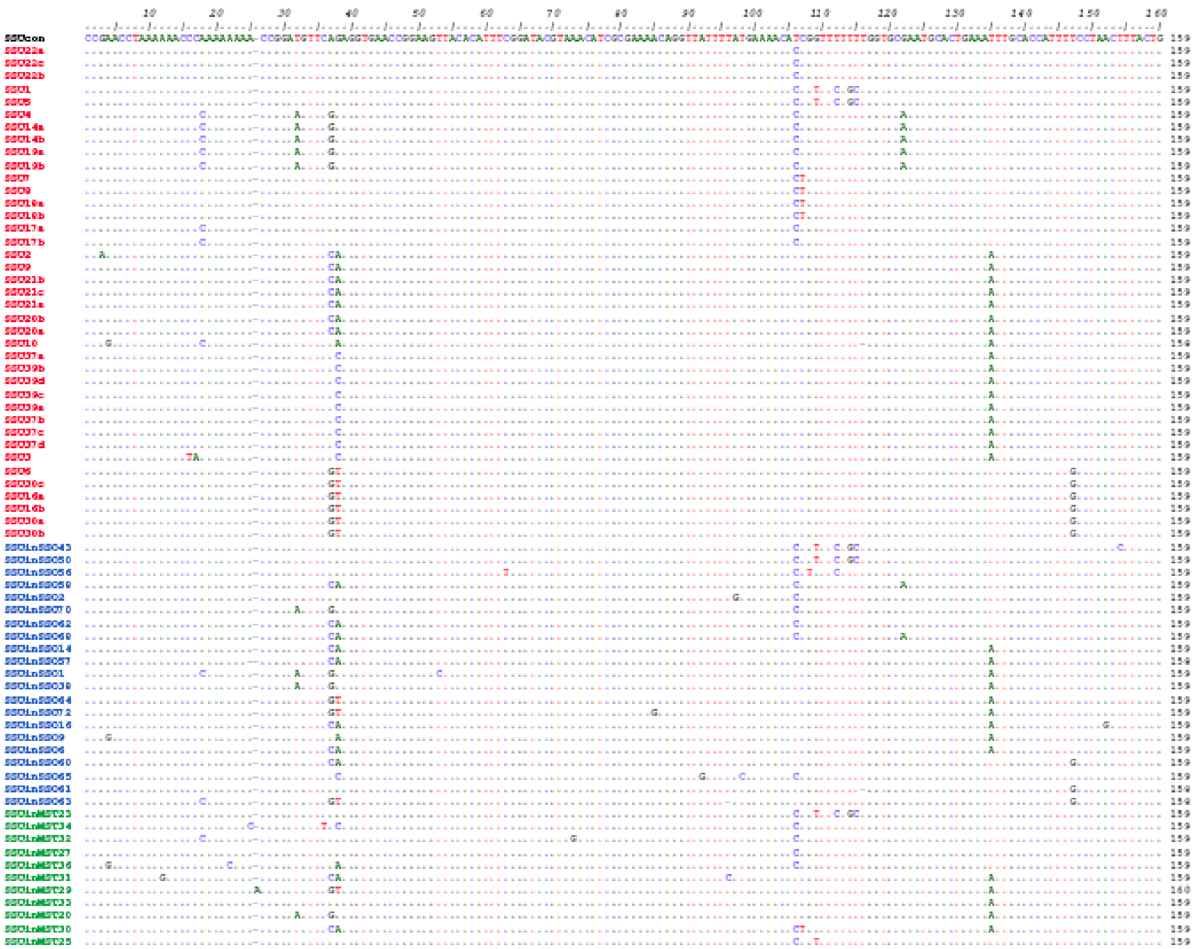

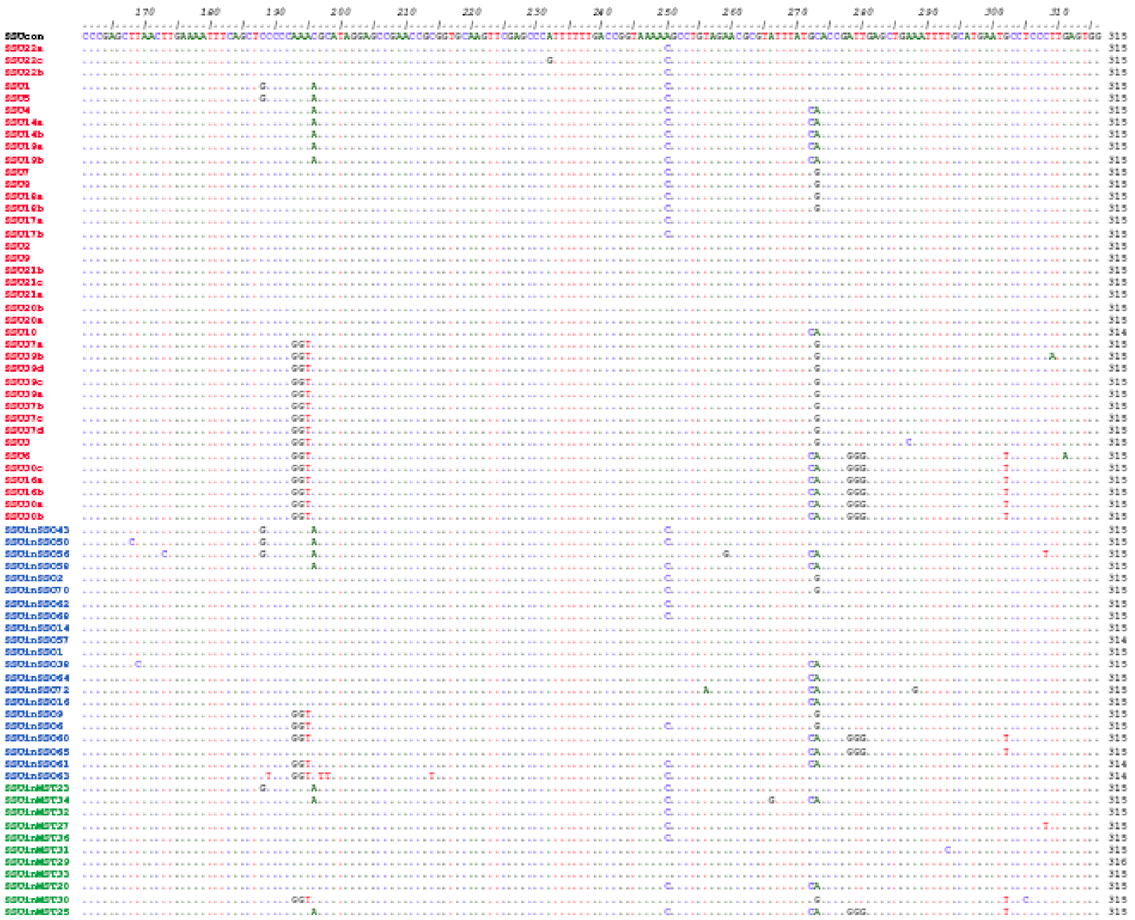

Supplementary Figure 2. Nucleotide alignment of SSUsat.

Nucleotide alignment of SSUsat nucleotide sequences isolated from *Spisula subtruncata* (**SSU**), *Spisula solida* (**SSUinSSO**) and *Mactra stultorum* (**SSUinMST**). Points and dashes indicate identical nucleotides and deletions, respectively.

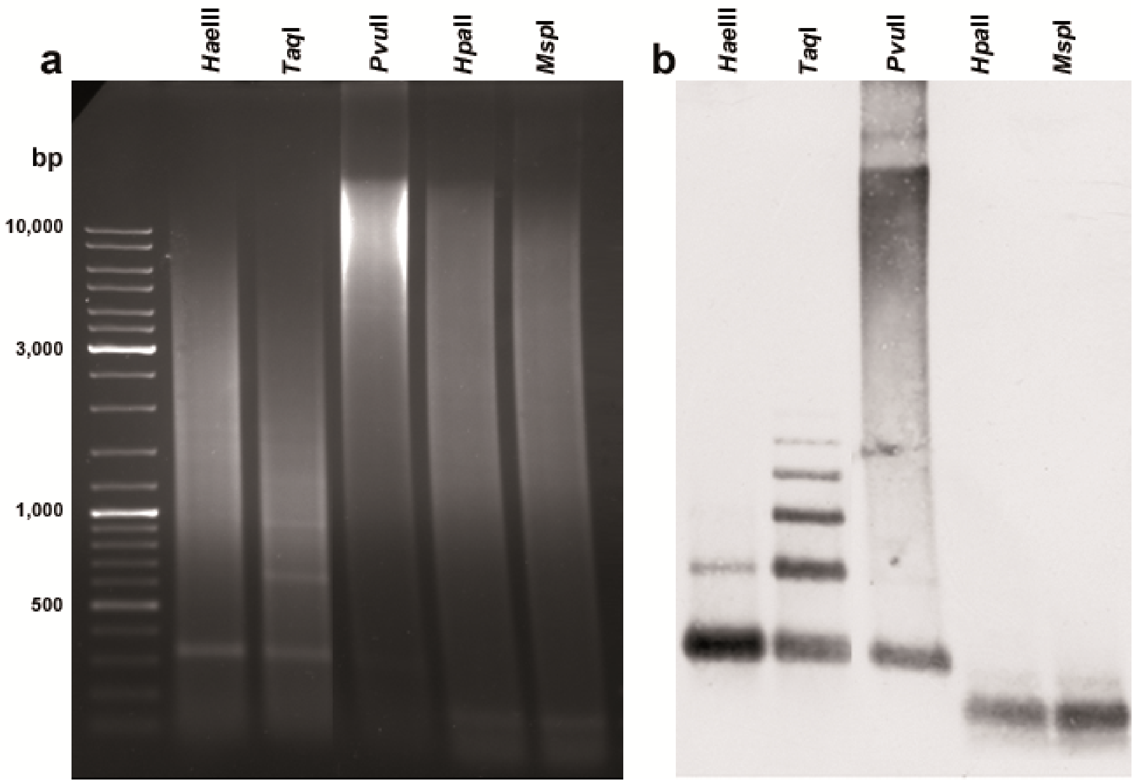

Supplementary Figure 3. Southern blotting of *Spisula subtruncata* genomic DNA digested with different REs.

Agarose gel electrophoresis of *Spisula subtruncata* genomic DNA digested either with REs that have single cleavage sites in conserved (*Hae*III and *Taq*I) or variable (*Pvu*II) regions of the SSUsat, or with two isoschizomers (*Hpa*II and *Msp*I) with three restriction targets in the SSUsat monomer sequence (**a**). After being Southern blotted on a nitrocellulose membrane, the electrophoresed DNA was hybridised with an SSUsat monomer probe (**b**).

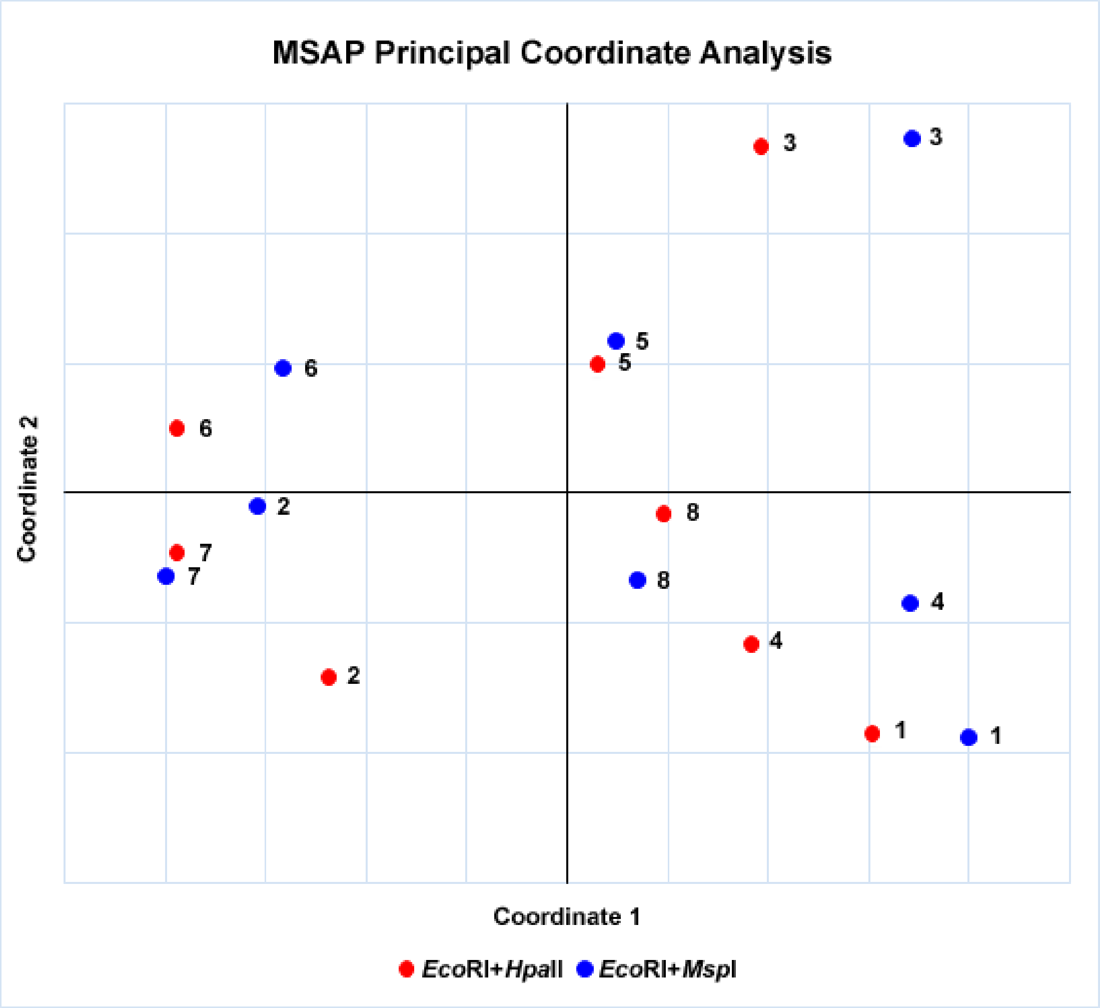

Supplementary Figure 4. MSAP principal coordinate analysis.

Principal coordinate analysis of the *Eco*RI+*Hpa*II (red) and *Eco*RI+*Msp*I (blue) restriction band profiles (MSAP) for eight *Spisula subtruncata* specimens (1 to 8) shows that the only clustering is between each pair of profiles from the same specimen, without any other clustering trend.

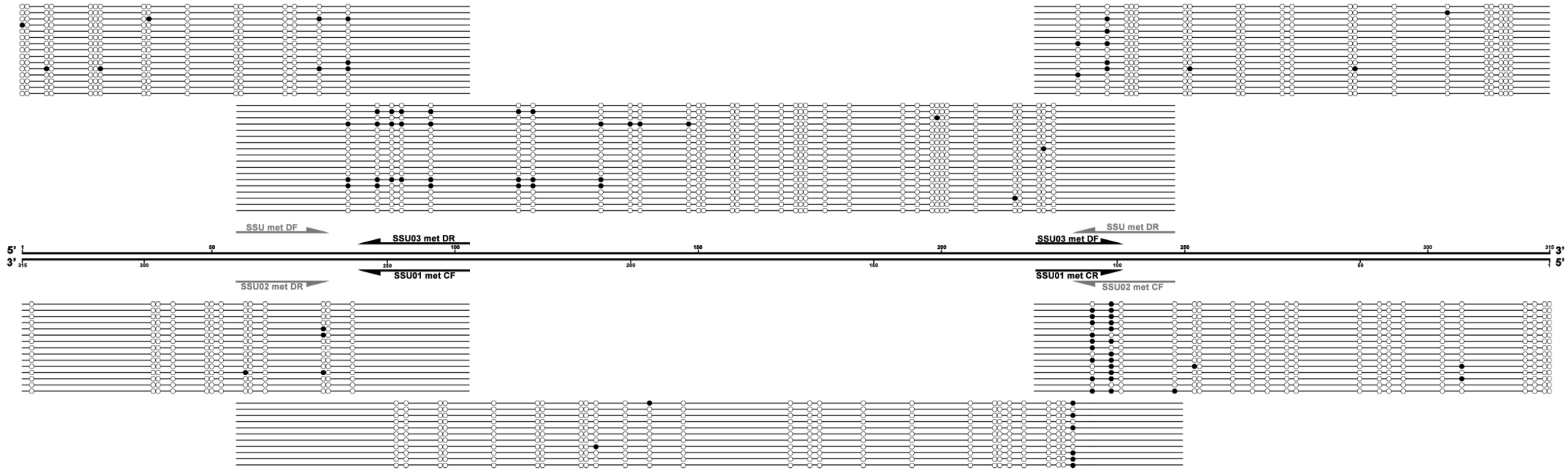

Supplementary Figure 5. Bisulphite analysis of *Spisula subtruncata* SSUsat.

Representative scaled lollipop diagrams for cytosine methylation of bisulfite transformed SSUsat clones. A total of 33 clones (15 external fragments and 18 internal) recovered for the direct strand (above the dsDNA) and 26 (15 external and 11 internal) for the complementary strand (below the dsDNA) were analysed. Bisulfite cloning and sequencing were carried out separately for both strands using four pairs of degenerated primers (indicated by grey and black arrows). Each horizontal line corresponds to an independently sequenced clone with cytosine methylation at CG, CHG, or CHH sites. Methylated cytosines are represented by filled circles, non-methylated cytosines appear as empty circles.
